# Phytosome Loading the Combined Extract of Mulberry Fruit and Ginger Protects against Cerebral Ischemia in Metabolic Syndrome Rats

**DOI:** 10.1155/2020/5305437

**Published:** 2020-07-25

**Authors:** Nut Palachai, Jintanaporn Wattanathorn, Supaporn Muchimapura, Wipawee Thukham-mee

**Affiliations:** ^1^Department of Physiology and Graduate School (Neuroscience Program), Faculty of Medicine, Khon Kaen University, Khon Kaen, Thailand 40002; ^2^Integrative Complementary Alternative Medicine Research and Development Center in Research Institute for Human High Performance and Health Promotion, Khon Kaen University, Khon Kaen, Thailand 40002; ^3^Department of Physiology, Faculty of Medicine, Khon Kaen University, Khon Kaen, Thailand 40002

## Abstract

The prevalence of ischemic stroke in metabolic syndrome (MetS) is continually increasing and produces a great impact on both qualities of life and annual healthcare budget. Due to the efficiency limitation of the current therapeutic strategy, the poor availability of polyphenol substances induced by the first pass effect and the beneficial effects of mulberry fruit and ginger on brain and MetS-related diseases together with the synergistic concept, the neuroprotective effect against ischemic stroke in MetS condition of phytosome containing the combined extract of mulberry fruit and ginger (PMG) has been considered. To explore the neuroprotective effect and possible underlying mechanism of PMG on brain damage in cerebral ischemic rat with MetS, male Wistar rats were induced MetS by high-carbohydrate high-fat diet (HCHF) for 16 weeks and subjected to the cerebral ischemia/reperfusion injury (CIRI) at the right middle cerebral artery (Rt. MCAO). PMG at doses of 50, 100, and 200 mg/kg were orally fed with for 21 days, and they were assessed brain damage, neurological deficit score, and the changes of oxidative stress markers, inflammatory markers, PPAR*γ* expression, and epigenetic modification via DNMT-1 were performed. All doses of PMG significantly improved brain infarction, brain edema, and neurological deficit score. In addition, the reduction in DNMT-1, MDA level, NF-*κ*B, TNF*α*, and C-reactive protein together with the increase in SOD, CAT, and GPH-Px activities, and PPAR*γ* expression in the lesion brain were also observed. The current data clearly revealed the neuroprotective effect against cerebral ischemia with MetS condition. The possible underlying mechanism might occur partly via the suppression of DNMT-1 giving rise to the improvement of signal transduction via PPAR*γ* resulting in the decreasing of inflammation and oxidative stress. In conclusion, PMG is the potential neuroprotectant candidate against ischemic stroke in the MetS condition. However, the clinical trial is still essential.

## 1. Introduction

Currently, stroke and metabolic syndrome (MetS) have been recognized as important health problems worldwide due to the increasing prevalence and the great impact on healthcare budget [1–3]. It has been demonstrated that MetS increases the risk of stroke [4–6] via the increase in intracranial atherosclerosis [7]. In addition, it can also increase oxidative stress and inflammation [8], the important factors playing the pivotal roles on the pathophysiology of ischemic stroke, and may increase severity of stroke [9]. Despite the increasing prevalence and importance of stroke with MetS, the therapeutic efficacy is not in the satisfaction level.

Recently, it has been demonstrated that the substance possessing antioxidant and anti-inflammation such as the combined extract of black stick rice and dill significantly improves brain damage and deficits in an animal model of ischemic stroke in MetS condition [10, 11]. Our previous study also clearly demonstrates that the phytosome containing the combined extract of ginger rhizome and mulberry fruit (PMG) can also decrease oxidative stress and inflammation [12]. This raises the possibility that PMG which also possesses the aforementioned activities [13–17] can also protect against ischemic stroke in MetS condition. Currently, no data concerning the aforementioned effect are available. Therefore, this study was set up to determine the neuroprotective effect against ischemic stroke in the rat model of MetS induced by a high carbohydrate high fat (HCHF) diet. In addition, the possible underlying mechanism especially the roles of oxidative stress, inflammation, PPAR*γ* signal transduction, and epigenetic mechanism modification were also determined.

## 2. Materials and Methods

### 2.1. Preparation of PMG

The authentication of ginger rhizomes (*Zingiber officinale* Roscoe) which were harvested from Khon Kaen province, Thailand, was performed by the expert in pharmacognosy of the National Museum of THAI Traditional Medicine, Thailand and the voucher specimen No. 0002402 was deposited at the National Museum of THAI Traditional Medicine. Ripen mulberry fruit (*Morus alba* Linn. var. Chiangmai) used in this study was identified and kindly provided by the Queen Sirikit Department of Sericulture Center (Udon Thani province), Ministry of Agriculture and Cooperatives, Thailand, and the voucher specimen 61001 was deposited at Research Institute of Human High Performance and Health Promotion, Khon Kaen University, Khon Kaen, Thailand. The phytosome loading with the combined extract of ginger rhizome extract and ripen mulberry fruit was prepared as prepared according to the method of Palachai and coworkers [12]. In brief, 50% and 95% hydroalcoholic extract of mulberry fruit and ginger were prepared by using the maceration method. Then, the combined extract at a ratio of 1 : 1 was mixed and encapsulated with phosphatidylcholine matrix in order to produce phytosome containing the combined extract of mulberry fruit and ginger (PMG). The concentrations of active ingredients including total phenolic compounds, flavonoids, gingerol, cyanidin-3-O-glucoside, quercetin-3-rutinoside, ferulic acid, and gallic acid in PMG were as same as those mentioned in our previous study [12].

### 2.2. Experimental Protocol

The experimental animals in this study were male Wistar rats, weighting 180-220 g (8 weeks old), from National Laboratory Animal Center, Salaya, Nakhon Pathom, Thailand. The animals were kept under standard laboratory conditions at 23 ± 2°C and 12 : 12 hours light-dark cycle. They were provided food and water ad libitum and housed in standard metal cages (6 per cage) and allowed to accustom to the laboratory condition for one week. All procedures and experimental protocols were approved by the Institutional Animal Ethics Committee of Khon Kaen University (record no. IACUC-KKU 95/60). After the acclimatization, all animals were randomly divided into 8 groups.

- Group I:Normal diet (ND) + vehicle: the animals in this group were provided a normal diet and treated with vehicle
- Group II:HCHF + Sham + vehicle: MetS rats induced by high-carbohydrate high-fat (HCHF) diet were orally given vehicle 21 days before and 14 days after exposing to sham operation
- Group III:HCHF + MCAO + vehicle: MetS rats induced by HCHF diet as mentioned in (b) were orally given vehicle 21 days before and 14 days after exposing to the occlusion of the right middle cerebral artery (Rt. MCAO).
- Group IV: HCHF + MCAO + vitamin C: MetS rats induced by HCHF diet and received vitamin C at a dose of 250 mg/kg BW 21 days before and 14 days after exposing to Rt. MCAO
- Group V: HCHF + MCAO + Piracetam: MetS rats induced by HCHF diet and received piracetam at a dose of 250 mg/kg BW 21 days before and 14 days after exposing to Rt. MCAO
- Group VI-VIII: HCHF + MCAO +PMG: MetS rats induced by HCHF diet and received PMG at various doses ranging from 50, 100, and 200 mg/kg BW 21 days before and 14 days after exposing to Rt. MCAO

**Figure 1:**
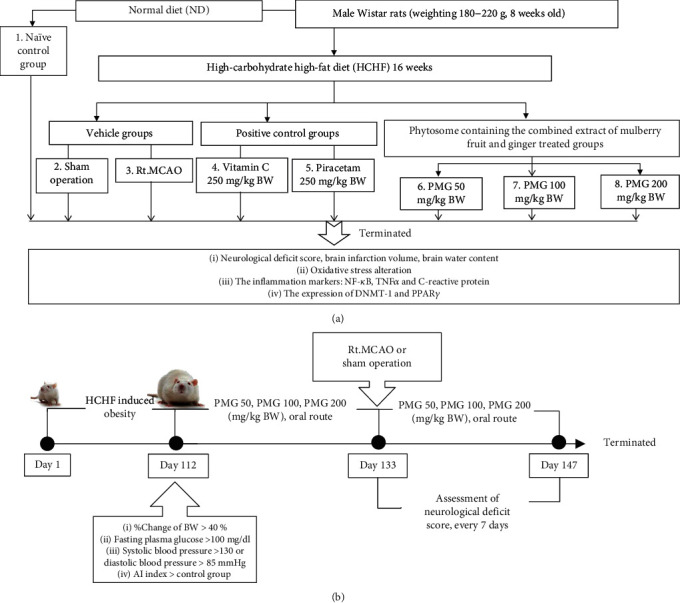
Schematic diagram showing all experimental procedures. (a) Experimental protocol of PMG treatment and the determination of various parameters. (b) Rt. MCAO induction and schedule for PMG treatment. Rt. MCAO: right middle cerebral artery occlusion; NF-*κ*B: nuclear factor-kappa-B; TNF*α*: tumor necrosis factor-alpha; DNMT-1: DNA methyltransferase-1; PPAR*γ*: peroxisome proliferator-activated receptor gamma; PMG 50, PMG 100, and PMG 200: the phytosome containing the combined extract of mulberry fruit and ginger at doses of 50, 100, and 200 mg.kg^−1^ BW, respectively.

The normal diet used in this study contained 4.5% fat, 42% carbohydrate, and 24% protein whereas the HCHF diet contained 35% fat, 45% carbohydrate, and 20% protein. After the exposure to a 16-week-feeding period, rats which showed the full criteria of MetS as mentioned in the previous study [12] were recruited for further study. The recruited animals were randomly assigned to various treatments as mentioned earlier. Food, water intake, and body weight changes were recorded every week. Then, 21 days after treatment with the assigned substances, the MetS rats were subjected to the occlusion of the right middle cerebral artery and continually treated with the assigned substances for 14 days after Rt. MCAO induction. The neurological deficit scores were assessed at 24 hours after Rt. MCAO and every 7 days throughout the study period while brain infarction volume, brain edema, the determinations C-reactive protein in serum were determined at 24 hours after Rt. MCAO. The determination of oxidative stress markers including malondialdehyde (MDA), superoxide dismutase (SOD), catalase (CAT), glutathione peroxidase (GSH-Px), and the expressions of DNA-methyltransferase 1 (DNMT-1), peroxisome proliferator-activated receptor-gamma (PPAR*γ*), nuclear factor kappa B (NF-*κ*B), and tumor necrosis factor-alpha (TNF*α*) in the lesioned brain were performed at the end of the study. The experimental protocol was summarized in Figure 1.

### 2.3. Induction of Right Middle Cerebral Artery Occlusion

Focal cerebral ischemia was induced by using ischemia/reperfusion (I/R) induction technique. After the anesthetization with pentobarbital sodium at a dose of 60 mg/kg BW, the nylon monofilament coated with silicone was inserted from the right common carotid artery into the internal carotid artery and put advanced forward until felt the faint resistance, toward the junction of anterior and middle cerebral arteries. After the exposure to a 90-minute Rt. MCAO, the filament was withdrawn to allow reperfusion and wound was sutured [18].

### 2.4. Neurological Score Assessment

To assess the functional consequences of cerebral ischemia, an 18-scale-modified neurologic severity score (mNSS) was used as a tool for monitoring neurological deficit [19]. According to this assessment, various components including motor (muscle status and abnormal movement), sensory (visual, tactile, and proprioceptive), reflex, and balance tests were evaluated. One point was given for the inability to perform each test, and an overall composite score was given to determine the neurological impairment. The details of grading scores assessed via an 18-scale-modified neurologic severity score (mNSS) test were provided in Table 1.

### 2.5. Brain Infarction Volume Assessment

Rat brains were removed and prepared as coronal sections at 2 mm thick. The brains were immediately incubated in 2% 2,3,5-triphenyltetrazolium chloride (TTC) solution (Sigma-Aldrich, St. Louis, MO, USA) in the dark at room temperature for 15 minutes and turned over every 5 min. Then, all brain slices were captured using a digital camera (Sony HDR-SR11 Handycam Camcorder; Sony Co. Ltd., Japan), and the brain infarction volume in cortex, striatum, and hippocampus was calculated with the computerized image analysis system [10]. The areas of the cerebral cortex, the outer layer covering all infrastructures, and the main subcortical structures such as the hippocampus and striatum were measured and calculated for the brain infarcted volume.

### 2.6. Brain Edema Assessment

Brain water content was measured by the standard wet–dry method [19]. After 24 hours of reperfusion, the sacrifice and brain isolation were carried out. The left and right hemispheres were weighed (wet weight) and were dried at 100°C for 24 hours to determine the brain dry weight. The degree of brain edema was calculated by the following equation:

(1) $$\begin{array}{c} \text{water content}=\frac{\;\left(\text{wet weight}-\text{dry weight}\right)}{\text{wet}}\text{weight}\times 100\%. \end{array}$$

### 2.7. Assessment of Oxidative Stress Status

The ipsilateral cortex was isolated and homogenized with 0.1 M potassium phosphate buffer solution in a ratio of 10 mg: 50 *μ*l. The brain tissue homogenate was used for determining oxidative stress status including malonaldehyde (MDA) level and the scavenging enzyme activities including superoxide dismutase (SOD), catalase (CAT), and glutathione peroxidase (GSH-Px). The protein concentrations in brain tissue homogenate were assessed by using a Thermo Scientific NanoDrop 2000c spectrophotometer (Thermo Fisher Scientific, Wilmington, Delaware, USA), and the optical density was measured at the wavelength of 280 nm.

MDA level, a lipid peroxidation product, was monitored by using thiobarbituric acid (TBA) reaction [20]. The reaction mixture containing 50 *μ*l of brain tissue homogenate, 50 *μ*l of 8.1% sodium dodecyl sulphate (SDS) (Sigma-Aldrich, USA), 375 *μ*l of 0.8% of thiobarbituric acid (TBA) (Sigma-Aldrich, USA), 375 *μ*l of 20% acetic acid (Sigma-Aldrich, USA), and 150 *μ*l of distilled water (DW) was boiled at 95°C in the water bath for 60 minutes. After incubation, it was cooled with tap water. Then, 250 *μ*l of distilled water and 1,250 *μ*l of the solution containing n-butanol and pyridine (Merck, Germany) at the ratio of 15 : 1 was added, mixed together, and centrifuged at 4,000 rpm for 10 minutes. The upper layer was separated and measured the absorbance at 532 nm. The standard curve was prepared by using TMP (1,1,3,3-tetramethoxy propane) at the concentrations of 0-15 *μ*M as reference (Sigma-Aldrich, USA). MDA level was expressed as ng/mg protein.

SOD activity determination was performed according to the method of Sun et al. [21]. In brief, 20 *μ*l of brain tissue homogenate was mixed with 200 *μ*l of the reaction mixture containing 57 mM phosphate buffer solution (KH_2_PO_4_) (Sigma-Aldrich, USA), 0.1 mM EDTA (Sigma-Aldrich, USA), 10 mM cytochrome C (Sigma-Aldrich, USA) solution, 50 *μ*M of xanthine (Sigma-Aldrich, USA), and 20 *μ*l of xanthine oxidase (0.90 mU/ml) (Sigma-Aldrich, USA). The absorbance at 415 nm was recorded. SOD enzyme (Sigma-Aldrich, USA) activities at the concentrations of 0-25 units/ml were served as standard, and the results were expressed as units/mg protein.

CAT activity was monitored based on the ability of the enzyme to break down H_2_O_2_. In brief, 10 *μ*l of brain tissue homogenate was mixed with the reaction mixture containing 50 *μ*l of 30 mM hydrogen peroxide (in 50 mM phosphate buffer, pH 7.0) (BDH Chemicals Ltd, UK), 25 *μ*l of 4 M H_2_SO_4_ (Sigma-Aldrich, USA), and 150 *μ*l of 5 mM KMnO_4_ (Sigma-Aldrich, USA). Following this process, an absorbance at 490 nm was measured [22]. CAT enzyme (Sigma-Aldrich, USA) at the concentration range between 10-100 units/ml was served as standard, and the result was expressed as units/mg protein.

GSH-Px activity was also determined. In brief, a mixture containing 20 *μ*l of brain tissue homogenate was mixed thoroughly with the reaction mixture consisting of 10 *μ*l of 1 mM dithiothreitol (DTT) (Sigma-Aldrich, USA) in 6.67 mM potassium phosphate buffer (pH 7.0), 100 *μ*l of 1 mM sodium azide (Sigma-Aldrich, USA) in 6.67 mM potassium phosphate buffer (pH 7.0), 10 *μ*l of 50 mM glutathione (Sigma-Aldrich, USA) solution, and 100 *μ*l of 30% hydrogen peroxide (BDH Chemicals Ltd, UK), and incubated at 25°C for 5 minutes. At the end of the incubation period, 10 *μ*l of 10 mM DTNB (5,5-dithiobis-2-nitrobenzoic acid) (Sigma-Aldrich, USA) was added and an absorbance at 412 nm was recorded at 25°C over a period of 5 min [23]. Various concentrations of GSH-Px enzyme (Sigma-Aldrich, USA) between 1-5 units/ml were served as standard and the result was expressed as units/mg protein.

### 2.8. Western Blotting Analysis

The ipsilateral cortex was homogenized in mammalian protein extraction reagent (M-PER; Pierce Protein Biology Product, Rockford, IL, USA), with protease inhibitor cocktail (1 : 10) (Sigma-Aldrich, USA) for assessing PPAR*γ*, NF-*κ*B, and TNF*α* expressions. In addition, an ipsilateral cortex was also homogenized and lysed in RIPA (radioimmunoprecipitation assay) buffer (Cell Signaling Technology, USA) containing 20 mM Tris-HCl (pH 7.5), 150 mM NaCl, 1 mM Na_2_EDTA, 1 mM EGTA, 1% NP-40, 1% sodium deoxycholate, 2.5 mM sodium pyrophosphate, 1 mM beta-glycerophosphate, 1 mM Na_3_VO_4_, 1 *μ*g/ml leupeptin, and 1 mM phenylmethanesulfonyl fluoride (PMSF) (Cell Signaling Technology, USA) for the determination of DNMT-1. Brain homogenates prepared according to the methods just mentioned were subjected to a 12,000 g-centrifugation process at 4°C for 10 minutes. The supernatant was isolated and used for the determination of protein and the expressions of DNMT-1, NF-*κ*B, TNF*α*, and PPAR*γ*. Protein concentration was determined by using a Thermo Scientific NanoDrop 2000c spectrophotometer (Thermo Fisher Scientific, Wilmington, DE, USA). In addition, an aliquot of sixty micrograms of brain tissue lysates were adjusted to an appropriate concentration by using Tris-Glycine SDS-PAGE loading buffer (Bio-Rad, USA) and heated at 95°C for 10 minutes. Protein in tissue sample was isolated via sodium dodecyl sulfate-polyacrylamide gel electrophoresis (SDS-PAGE) by loading 20 *μ*l of sample on SDS-polyacrylamide gel. Then, the separated bands were transferred to nitrocellulose membrane, washed with 0.05% TBS-T, and incubated in blocking buffer (5% skim milk in 0.1% TBS-T) at 25°C for 1 hour. After the blocking process, the nitrocellulose membrane was incubated with anti-DNMT-1 (Cell Signaling Technology, USA; dilution 1 : 1000), anti-NF-*κ*B p65 (Cell Signaling Technology, USA; dilution 1 : 500), anti-TNF*α* (Cell Signaling Technology, USA; dilution 1 : 1000), anti-PPAR*γ* (Cell Signaling Technology, USA; dilution 1 : 1000), and anti-*β* actin (Cell Signaling Technology, USA; dilution 1 : 1000) antibodies at 25°C for 2 hours. The nitrocellulose membrane was rinsed with TBS-T (0.05%) again and incubated with anti-rabbit IgG, HRP-linked antibody (Cell Signaling Technology, USA; dilution 1 : 2000) at 25°C for 1 hour. The bands were visualized and quantitated by using the ECL detection systems (GE Healthcare) and LAS-4000 luminescent image analyzer (GE Healthcare). Band intensities were measured using ImageQuant TL v.7.0 image analysis software (GE Healthcare). The expressions of DNMT-1, NF-*κ*B, TNF*α*, and PPAR*γ* were normalized using anti-*β* actin [12, 24]. Data were presented as a relative density to the naïve control group.

### 2.9. Statistical Analysis

All data are expressed as mean ± standard error of mean (SEM). Statistical significance was evaluated by using a one-way analysis of variance (ANOVA), followed by the post hoc (Tukey) test. Student's *t* test was used for comparing the means for two groups. Statistical significance was regarded at *p* value < 0.05. All statistical data analyses were performed using SPSS version 21.0 (IBM Corp. Released 2012. IBM SPSS Statistics for Windows).

## 3. Results

### 3.1. Effect of PMG on Brain Damage

**Figure 2:**
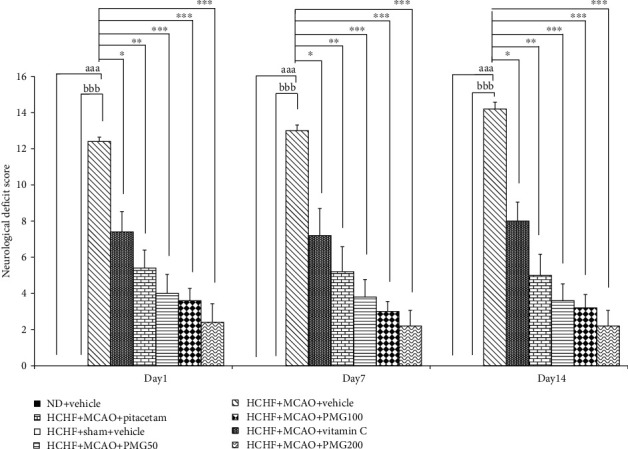
Effect of various doses of PMG on neurological deficit score. Data are presented as mean ± SEM (*n* = 6/group). ^aaa^*p* values < 0.001; compared between naïve control rats which received normal diet and vehicle, and metabolic syndrome+MCAO rats which received HCHF, MCAO, and vehicle, ^bbb^*p* values < 0.001; compared between sham rats which received HCHF, sham operation, and vehicle, and metabolic syndrome+MCAO rats which received HCHF, MCAO, and vehicle and ^∗^, ^∗∗^, ^∗∗∗^*p* values < 0.05, 0.01, and 0.001, respectively; compared to metabolic syndrome+MCAO rats which received HCHF, MCAO, and vehicle. ND: normal diet; HCHF: high-carbohydrate high-fat diet: MCAO: middle cerebral artery occlusion; vitamin C: the vitamin C at a dose of 250 mg.kg^−1^ BW; piracetam: the piracetam at a dose of 250 mg.kg^−1^ BW; PMG 50, PMG 100, and PMG 200: the phytosome containing the combined extract of mulberry fruit and ginger at doses of 50, 100, and 200 mg.kg^−1^ BW, respectively.

The effect of PMG on the neurological deficit score was shown in Figure 2. The results showed that the MetS rats which received MCAO and vehicle showed the significant increase in neurological deficit score at 1, 7, and 14 days after MCAO (*p* values < 0.001 all; compared to the HCHF+sham operation+vehicle group), whereas sham operation failed to produce neurological deficit. However, the changes induced by MCAO were counteracted by all doses of PMG at 1, 7, and 14 days after MCAO (*p* values < 0.001 all; compared to the HCHF+MCAO+vehicle group). In addition, both piracetam and vitamin C also significantly improved neurological deficit score at 1, 7, and 14 days after MCAO (*p* values < 0.05 and 0.01, respectively; compared to the HCHF+MCAO+vehicle group).

**Figure 3:**
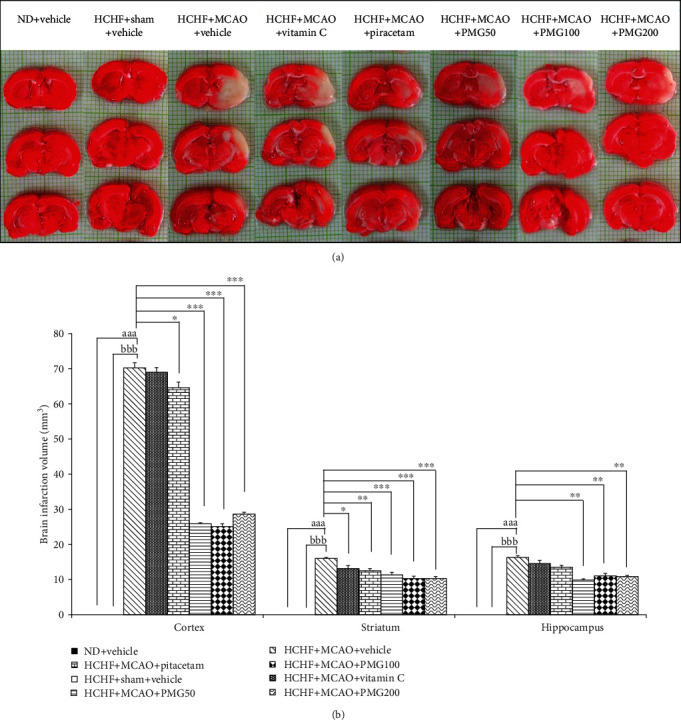
Effect of various doses of PMG on brain infarction volume. (a) The photograph of coronal sections of the brains which were stained with 2,3,5-triphenyltetrazolium chloride (TTC). (b) Brain infarction volume in cortex, striatum, and hippocampus. Data are presented as mean ± SEM (*n* = 6/group). ^aaa^*p* values < 0.001; compared between naïve control rats which received normal diet and vehicle, and metabolic syndrome+MCAO rats which received HCHF, MCAO, and vehicle, ^bbb^*p* values < 0.001; compared between sham rats which received HCHF, sham operation, and vehicle, and metabolic syndrome+MCAO rats which received HCHF, MCAO, and vehicle and ^∗^, ^∗∗^, ^∗∗∗^*p* values < 0.05, 0.01, and 0.001, respectively; compared to metabolic syndrome+MCAO rats which received HCHF, MCAO, and vehicle. ND: normal diet; HCHF: high-carbohydrate high-fat diet; MCAO: middle cerebral artery occlusion; vitamin C: the vitamin C at a dose of 250 mg.kg^−1^ BW; piracetam: the piracetam at a dose of 250 mg.kg^−1^ BW; PMG 50, PMG 100, and PMG 200: the phytosome containing the combined extract of mulberry fruit and ginger at doses of 50, 100, and 200 mg.kg^−1^ BW, respectively.

Figure 3 showed that the MetS rats which received sham operation failed to produce brain infarction, whereas MCAO and vehicle significantly increased brain infarction volume in cortex, striatum, and hippocampus (*p* values < 0.001 all; compared to the HCHF+sham operation+vehicle group). Piracetam treatment significantly improved brain infarction in cortex and striatum (*p* values < 0.05 and 0.01, respectively; compared to the HCHF+MCAO+vehicle group), whereas vitamin C significantly attenuated brain infarction only in striatum (*p* values < 0.05; compared to the HCHF+MCAO+vehicle group). Interestingly, all doses of PMG produced the significant reduction in brain infarction volume in the cortex, striatum, and hippocampus (*p* values < 0.001 all; *p* values < 0.001 all; *p* values < 0.01 all; compared to the HCHF+MCAO+vehicle group).

**Figure 4:**
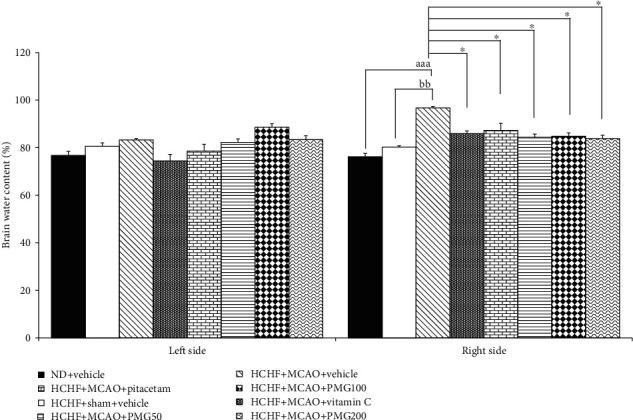
Effect of various doses of PMG on brain water content. Data are presented as mean ± SEM (*n* = 6/group). ^aaa^*p* values < 0.001; compared between naïve control rats which received a normal diet, and vehicle, and metabolic syndrome+MCAO rats which received HCHF, MCAO, and vehicle, ^bb^*p* values < 0.01; compared between sham rats which received HCHF, sham operation, and vehicle, and metabolic syndrome+MCAO rats which received HCHF, MCAO, and vehicle and ^∗^*p* values < 0.05; compared to metabolic syndrome+MCAO rats which received HCHF, MCAO, and vehicle. ND: normal diet; HCHF: high-carbohydrate high-fat diet; MCAO: middle cerebral artery occlusion; vitamin C: the vitamin C at a dose of 250 mg.kg^−1^ BW; piracetam: the piracetam at a dose of 250 mg.kg^−1^ BW; PMG 50, PMG 100, and PMG 200: the phytosome containing the combined extract of mulberry fruit and ginger at doses of 50, 100, and 200 mg.kg^−1^ BW, respectively.

The effect of PMG on brain edema was shown in Figure 4. The results revealed that the contralateral hemisphere of MetS rats which subjected to I/R failed to produce the significant changes of brain water content. However, the significant change in brain water content was observed in the ipsilateral hemisphere of MetS rats which received MCAO and vehicle (*p* values < 0.01; compared to the HCHF+sham operation+vehicle group). All doses of PMG, vitamin C, and Piracetam treated groups significantly decreased brain water content (*p* values < 0.05 all; compared to the HCHF+MCAO+vehicle group).

### 3.2. Effect of PMG on Oxidative Stress Status

Table 2 demonstrated the effect of PMG on oxidative stress status in the cerebral cortex including the level of MDA and the activities of the main scavenger enzymes including SOD, CAT, and GSH-Px. It was found that sham operation failed to show the significant change in oxidative stress status. MetS rats which subjected to MCAO and received vehicle significantly decreased SOD, CAT, and GSH-Px activities (*p* values < 0.001 all; compared to normal diet group and *p* values < 0.001 all; compared to the HCHF+sham operation+vehicle group). MetS rats which subjected to MCAO and received vitamin C significantly increased SOD and GSH-Px activities but decreased MDA level (*p* values < 0.01, 0.05, and 0.05, respectively; compared to the HCHF+MCAO+vehicle group), whereas piracetam failed to produce the significant changes on aforementioned parameters. PMG treatment at doses of 50 and 100 mg/kg also produced the significant decrease in MDA level (*p* values < 0.05 all; compared to the HCHF+MCAO+vehicle group) together with the significant increase in SOD (*p* values < 0.05 and 0.001, respectively; compared to the HCHF+MCAO+vehicle group), CAT (*p* values < 0.01 all; compared to the HCHF+MCAO+vehicle group), and GSH-Px activities (*p* values < 0.05 and 0.001, respectively; compared to the HCHF+MCAO+vehicle group) in the cerebral cortex of MetS rats with MCAO. In addition, PMG treatment at a dose of 200 mg/kg also significantly decreased MDA but increased SOD and GSH-Px (*p* values < 0.05, 0.01, and 0.05, respectively; compared to the HCHF+MCAO+vehicle group). However, no significant change of CAT was observed in MetS rats with cerebral ischemia and received the high dose of PMG.

### 3.3. Effect of PMG on Biochemical Changes

**Figure 5:**
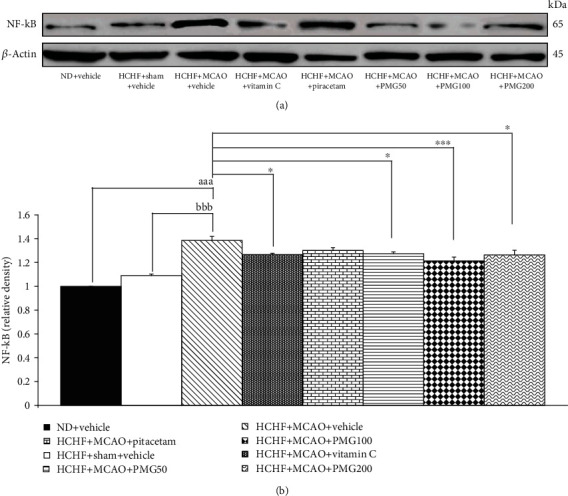
Effect of various doses of PMG on the expression of NF-*κ*B in the cerebral cortex was detected by Western blotting. (a) Representative western blot showing the levels of NF-*κ*B. (b) Relative density of NF-*κ*B. Data are presented as mean ± SEM (*n* = 6/group). ^aaa^*p* values < 0.001; compared between naïve control rats which received normal diet and vehicle, and metabolic syndrome+MCAO rats which received HCHF, MCAO, and vehicle, ^bbb^*p* values < 0.001; compared between sham rats which received HCHF, sham operation, and vehicle, and metabolic syndrome+MCAO rats which received HCHF, MCAO, and vehicle and ^∗^, ^∗∗∗^*p* values < 0.05 and 0.001, respectively; compared to metabolic syndrome+MCAO rats which received HCHF, MCAO, and vehicle. ND: normal diet; HCHF: high-carbohydrate high-fat diet; MCAO: middle cerebral artery occlusion; vitamin C: the vitamin C at a dose of 250 mg.kg^−1^ BW; piracetam: the piracetam at a dose of 250 mg.kg^−1^ BW; PMG 50, PMG 100, and PMG 200: the phytosome containing the combined extract of mulberry fruit and ginger at doses of 50, 100, and 200 mg.kg^−1^ BW, respectively.

**Figure 6:**
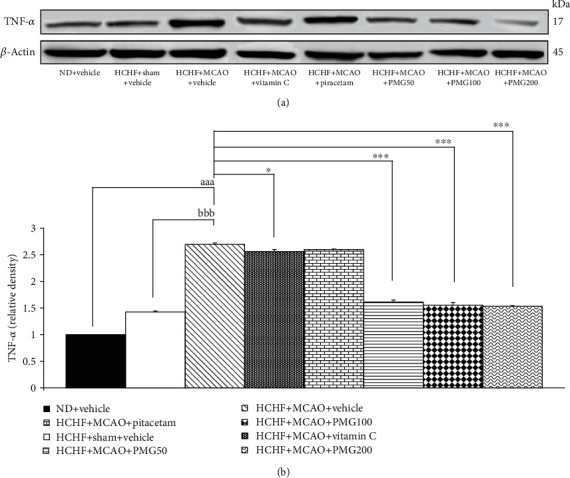
Effect of various doses of PMG on the expression of TNF-*α* in the cerebral cortex was detected by Western blotting. (a) Representative western blot showing the levels of TNF*α*. (b) Relative density of TNF*α*. Data are presented as mean ± SEM (*n* = 6/group). ^aaa^*p* values < 0.001; compared between naïve control rats which received normal diet and vehicle, and metabolic syndrome+MCAO rats which received HCHF, MCAO, and vehicle, ^bbb^*p* values < 0.001; compared between sham rats which received HCHF, sham operation, and vehicle, and metabolic syndrome+MCAO rats which received HCHF, MCAO, and vehicle and ^∗^, ^∗∗∗^*p* values < 0.05 and 0.001, respectively; compared to metabolic syndrome+MCAO rats which received HCHF, MCAO, and vehicle. ND: normal diet; HCHF: high-carbohydrate high-fat diet; MCAO: middle cerebral artery occlusion; vitamin C: the vitamin C at a dose of 250 mg.kg^−1^ BW; piracetam: the piracetam at a dose of 250 mg.kg^−1^ BW; PMG 50, PMG 100, and PMG 200: the phytosome containing the combined extract of mulberry fruit and ginger at doses of 50, 100, and 200 mg.kg^−1^ BW, respectively.

The effect of PMG on inflammatory markers such as NF-*κ*B, TNF*α* in cortex was assessed and the results were shown in Figures 5 and 6. It was found that MetS rats which subjected to sham operation failed to produce the significant change on the expression of NF-*κ*B in the cerebral cortex. MCAO significantly enhanced NF-*κ*B expression in the aforementioned area (*p* values < 0.001; compared to normal diet fed rats which received vehicle; *p* values < 0.001; compared to compared to the HCHF+sham operation+vehicle group). The elevation of NF-*κ*B expression was attenuated significantly by vitamin C and all doses of PMG (*p* values < 0.05, 0.05, 0.001, and 0.05, respectively; compared to the HCHF+MCAO+vehicle group). Piracetam failed to produce the significant change of this parameter.

The effect of PMG on TNF*α* expression in the cerebral cortex was shown in Figure 6. The similar pattern of changes was also observed. MetS rats which exposed to sham operation failed to produce the significant change on TNF*α* expression. However, MCAO produced a significant increase in TNF*α* expression (*p* values < 0.001; compared to normal diet-fed rats which received vehicle; *p* values < 0.001; compared to compared to the HCHF+sham operation+vehicle group). This elevation was counteracted by vitamin C treatment (*p* values < 0.05; compared to the HCHF+MCAO+vehicle group). In addition, all doses of PMG also significantly decreased TNF*α* expression (*p* values < 0.001 all; compared to the HCHF+MCAO+vehicle group).

**Figure 7:**
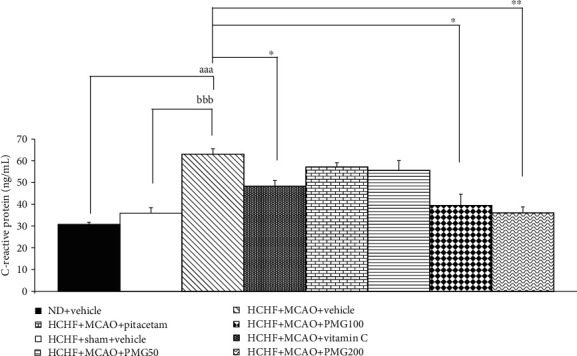
Effect of various doses of PMG on C-reactive protein in serum. Data are presented as mean ± SEM (*n* = 6/group). ^aaa^*p* values < 0.001; compared between naïve control which received normal diet and vehicle, and metabolic syndrome+MCAO rats which received HCHF, MCAO, and vehicle, ^bbb^*p* values < 0.001; compared between sham rats which received HCHF, sham operation, and vehicle, and metabolic syndrome+MCAO rats which received HCHF, MCAO, and vehicle and ^∗^, ^∗∗^*p* values < 0.05 and 0.01, respectively; compared to metabolic syndrome+MCAO rats which received HCHF, MCAO, and vehicle. ND: normal diet; HCHF: high-carbohydrate high-fat diet; MCAO: middle cerebral artery occlusion; vitamin C: the vitamin C at a dose of 250 mg.kg^−1^ BW; piracetam: the piracetam at a dose of 250 mg.kg^−1^ BW; PMG 50, PMG 100, and PMG 200: the phytosome containing the combined extract of mulberry fruit and ginger at doses of 50, 100, and 200 mg.kg^−1^ BW, respectively.

The effect of PMG on serum C-reactive protein level was also investigated and results were shown in Figure 7. MetS rats which subjected to sham operation failed to produce the significant modulation on this parameter whereas MCAO significantly enhanced C-reactive protein level in serum of MetS rats (*p* values < 0.001; compared to normal diet-fed rats which received vehicle; *p* values < 0.001; compared to compared to the HCHF+sham operation+vehicle group). These changes were attenuated by vitamin C and both medium and high doses of PMG (*p* values <0.05, 0.05, and 0.01, respectively; compared to the HCHF+MCAO+vehicle group).

**Figure 8:**
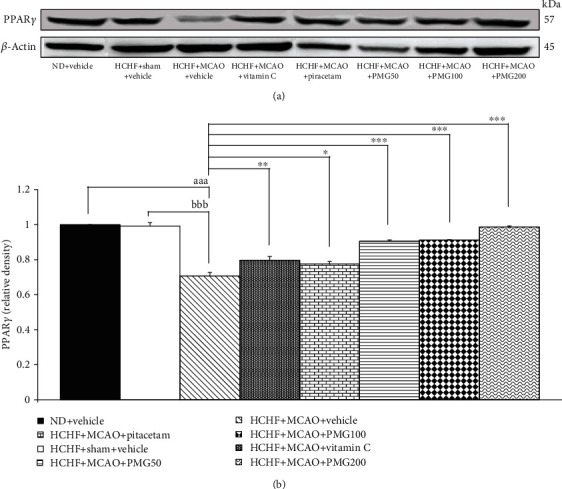
Effect of various doses of PMG on the expression of PPAR*γ* in the cerebral cortex was detected by Western blotting. (a) Representative western blot showing the levels of PPAR*γ*. (b) Relative density of PPAR*γ*. Data are presented as mean ± SEM (*n* = 6/group). ^aaa^*p* values < 0.001; compared between naïve control rats which received normal diet and vehicle, and metabolic syndrome+MCAO rats which received HCHF, MCAO, and vehicle, ^bbb^*p* values < 0.001; compared between sham rats which received HCHF, sham operation, and vehicle, and metabolic syndrome+MCAO rats which received HCHF, MCAO, and vehicle and ^∗^, ^∗∗^, ^∗∗∗^*p* values < 0.05, 0.01, and 0.001, respectively; compared to metabolic syndrome+MCAO rats which received HCHF, MCAO, and vehicle. ND: normal diet; HCHF: high-carbohydrate high-fat diet; MCAO: middle cerebral artery occlusion; vitamin C: the vitamin C at a dose of 250 mg.kg^−1^ BW; piracetam: the piracetam at a dose of 250 mg.kg^−1^ BW; PMG 50, PMG 100, and PMG 200: the phytosome containing the combined extract of mulberry fruit and ginger at doses of 50, 100, and 200 mg.kg^−1^ BW, respectively.

The effect of PMG on PPAR*γ* expression in the cerebral cortex was shown in Figure 8. It was found that MetS rats induced by HCHF diet which subjected to sham operation failed to produce the significant modulation on PPAR*γ* expression, whereas MCAO significantly decreased PPAR*γ* expression in the cerebral cortex of MetS rats (*p* values < 0.001; compared to normal diet-fed rats which received vehicle; *p* values < 0.001; compared to compared to the HCHF+sham operation+vehicle group). The decrease in PPAR*γ* expression significantly increased by vitamin C, piracetam, and all doses of PMG (*p* values < 0.01, 0.05, 0.001, 0.001, and 0.001, respectively; compared to the HCHF+MCAO+vehicle group).

**Figure 9:**
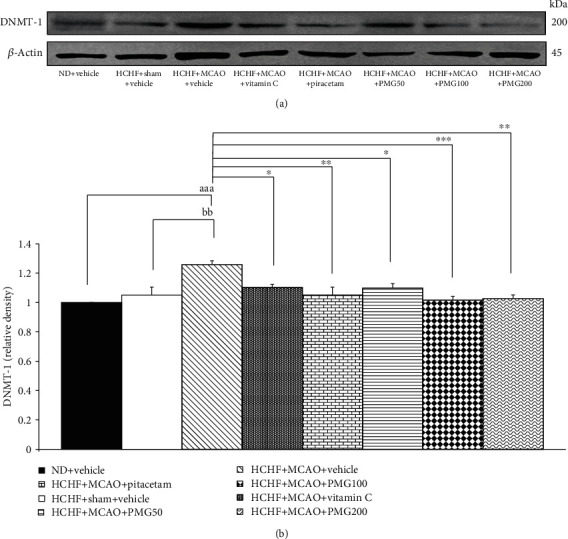
Effect of various doses of PMG on the expression of DNMT-1 in the cerebral cortex was detected by Western blotting. (a) Representative western blot showing the levels of DNMT-1. (b) Relative density of DNMT-1. Data are presented as mean ± SEM (*n* = 6/group). ^aaa^*p* values < 0.001; compared between naïve control rats which received normal diet and vehicle, and metabolic syndrome+MCAO rats which received HCHF, MCAO, and vehicle, ^bb^*p* values < 0.01; compared between sham rats which received HCHF, sham operation, and vehicle, and metabolic syndrome+MCAO rats which received HCHF, MCAO, and vehicle and ^∗^, ^∗∗^, ^∗∗∗^*p* values < 0.05, 0.01, and 0.001, respectively; compared to metabolic syndrome+MCAO rats which received HCHF, MCAO, and vehicle. ND: normal diet; HCHF: high-carbohydrate high-fat diet; MCAO: middle cerebral artery occlusion; vitamin C: the vitamin C at a dose of 250 mg.kg^−1^ BW; piracetam: the piracetam at a dose of 250 mg.kg^−1^ BW; PMG 50, PMG 100, and PMG 200: the phytosome containing the combined extract of mulberry fruit and ginger at doses of 50, 100, and 200 mg.kg^−1^ BW, respectively.

Figure 9 showed the modification effect of PMG on the epigenetic mechanism was assessed by using DNMT-1 as indicator. Sham operation failed to produce the significant modulation on DNMT-1 expression in the cerebral cortex of MetS rats induced by HCHF diet whereas MCAO significantly enhanced DNMT-1 expression in the cerebral cortex of MetS rats (*p* values < 0.001; compared to normal diet-fed rats which received vehicle; *p* values < 0.01; compared to compared to the HCHF+sham operation+vehicle group). The increase in DNMT-1 expression significantly decreased by vitamin C, piracetam, and all doses of PMG (*p* values < 0.05, 0.01, 0.05, 0.001, and 0.01, respectively; compared to the HCHF+MCAO+vehicle group).

## 4. Discussion

The current study has clearly demonstrated that the phytosome containing the combined extract of mulberry fruit and ginger significantly improves neurological deficit and brain damage in an animal model of stroke in MetS condition induced by the I/R injury at the middle cerebral artery in HCHF diet-fed rats. The oxidative stress status, inflammation, and PPAR*γ* together with DNMT-1 are also improved.

Our data have demonstrated that MetS rats with cerebral ischemia show the elevation of oxidative stress and inflammatory markers in the brain. These findings are corresponding with our previous study [11]. These changes give rise to brain damage which in turn induces the failure of ATPase pumps which resensitive to free radical reactions and lipid peroxidation. The impairment of ATPase pumps especially Na^+^-K^+^ ATPase triggers brain edema [25] which in turn significantly influences the degree of neurological deficit [26]. Based on these pieces of information, we suggest that the improvement of brain edema, brain damage, and neurological score deficit in this study may occur partly via the improvement of oxidative stress and inflammation. It has been revealed that both oxidative stress and inflammation are under the influence of PPAR*γ*. PPAR*γ* agonists suppress both processes and give rise to the neuroprotection against cerebral ischemia [27, 28]. Recent findings also reveal that the expression of PPAR*γ* is under the influence of epigenetic modification, the modification of the mediator between genetic and environment. DNA hypermethylation suppresses the expression of PPAR*γ* which in turn suppresses the anti-inflammatory cytokine production via the activation of M2 macrophages [29] which in turn increases inflammatory reaction, cell proliferation, and differentiation of foam cells leading to atherosclerosis and ischemic stroke [30, 31]. Therefore, the increase PPAR*γ* expression in this study may partly relate to the decrease of DNA methylation.

Interestingly, anthocyanins- rich substances reveal a significant epigenetic modification via both DNA methyltransferase-1 [32]. and histone deacetylase-3 [12]. In addition, many natural products such as ginger and anthocyanins- rich substance can also increase the expression of PPAR*γ* [33, 34]. These findings are corresponding with our data. It has been shown that PMG significantly decreases methylation of DNA via the reduction of DNMT-1 expression which in turn increases PPAR*γ* expression. Taken all data together, we suggest that PMG significantly suppresses the expression of DNMT-1 giving rise to the increase in PPAR*γ* expression leading to the decrease in inflammatory markers such as TNF*α*, NF-*κ*B, and C-reactive protein leading to the decrease of brain infarction and brain edema resulting in the improvement of neurological deficit. The increase in PPAR*γ* expression may also improve both parameters just mentioned either via the decrease in oxidative stress status [35] which in turn directly decreases both brain infarction and brain edema [36] or indirectly via the reduction of inflammation and results in the improvement of both brain infarction and brain edema as mentioned earlier. Since PMG also possesses both antioxidant and anti-inflammation [12], it may be possible to improve brain infarction and brain edema via direct its effect.

It has been revealed that both ginger extract and mulberry fruit extract can improve oxidative stress status by enhancing antioxidative enzymes giving rise to the reduction of brain damage and dysfunction [13, 14, 37]. Based on this information and the antioxidant and anti-inflammation of PMG mentioned earlier, we suggest that the neuroprotective effect of PMG observed in this study may be associated with the combined extract of mulberry fruit and ginger of PMG.

## 5. Conclusion

**Figure 10:**
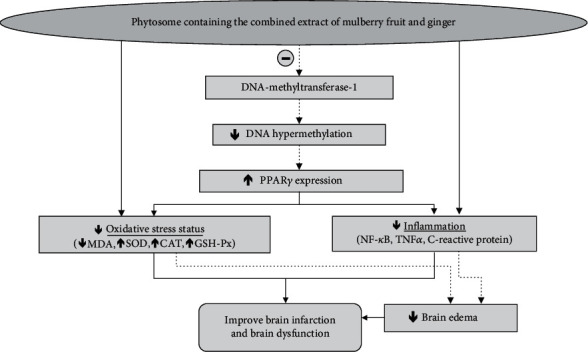
The schematic diagram demonstrated the positive modulation effect of PMG in the animal model of cerebral ischemia in metabolic syndrome condition. PPAR*γ*: peroxisome proliferator-activated receptor gamma; NK-*κ*B: nuclear factor kappa B; TNF*α*: tumor necrosis factor-alpha; MDA: malondialdehyde; SOD: superoxide dismutase; CAT: catalase; GSH-Px: glutathione peroxidase.

The present study has demonstrated that PMG is the potential candidate to protect against brain damage and dysfunction induced by the occlusion of the middle cerebral artery in MetS condition. PMG can produce the direct suppression effect on oxidative stress and inflammation. In addition, it can increase antioxidant enzymes which in turn decrease oxidative stress status. Moreover, the decrease of both inflammation and oxidative stress status can also occur as the result of the suppression of DNMT-1 giving rise to the increase of PPAR*γ* expression leading to the decrease in oxidative stress and inflammation and finally result in the improvement of brain infarction and brain dysfunction. The improvement of brain edema appears to occur as the result of the improvement of inflammation either via the increased PPAR*γ* expression induced by the suppression of DNMT-1 mentioned earlier or via the reduction of oxidative stress as shown in Figure 10. However, further research in clinical trial study is very much essential to confirm this neuroprotective effect.

## Figures and Tables

**Table 1:** Criteria for assessing the neurological score assessment via an 18-scale- modified neurologic severity score (mNSS).

Behavioral test	Score
Motor tests (normal = 0, maximum = 6)	
Raising rat by tail (normal = 0, maximum = 3)	
-Flexion of fore limb	1
-Flexion of hind limb	1
-Head moved >10° to the vertical axis within 30 seconds	1
Placing rat on floor (normal = 0, maximum = 3)	
-Normal walk	0
-Inability to walk straight	1
-Circling toward the paretic side	1
-Falling down to the paretic side	1
Sensory tests (normal = 0, maximum = 2)	
-Placing test (visual and tactile test)	1
-Proprioceptive test (deep sensation, pushing paw against table edge to stimulate limb muscles)	1
Beam balance testes (normal = 0, maximum = 6)	
-Balance with steady posture	0
-Grasps side of beam	1
-Hugs beam and 1 limb falls down from beam	2
-Hugs beam and 2 limbs falls down from beam, or spins on beam (>60 s)	3
-Attempts to balance on beam but falls off (>40 s)	4
-Attempts to balance on beam but falls off (>20 s)	5
-Fall off, no attempt to balance or hang on the beam (<20 s)	6
Reflex absence and abnormal movements (normal = 0, maximum = 4)	
-Pinna reflex (head shake when auditory meatus is touched)	1
-Corneal reflex (eye blink when cornea is lightly touch with cotton)	1
-Startle reflex (motor response to a brief noise from clapping hands)	1
-Seizures, myoclonus, myodystony	1
Maximum points	18

**Table 2:** The effect of various doses of PMG on oxidative stress markers in the cerebral cortex.

Treatment groups	MDA (ng/mg protein)	SOD (units/mg protein)	CAT (units/mg protein)	GSH-Px (units/mg protein)
ND+vehicle	0.27 ± 0.04	6.76 ± 0.30	17.47 ± 0.76	8.22 ± 0.49
HCHF+sham+vehicle	0.29 ± 0.02	6.49 ± 0.14	16.85 ± 1.26	7.96 ± 0.64
HCHF+MCAO+vehicle	2.33 ± 0.25^aaa, bbb^	1.16 ± 0.24^aaa, bbb^	0.52 ± 0.09^aaa, bbb^	0.99 ± 0.16^aaa, bbb^
HCHF+MCAO+vitamin C	0.52 ± 0.04^∗^	4.38 ± 0.35^∗∗^	6.99 ± 0.61	5.79 ± 0.36^∗^
HCHF+MCAO+Piracetam	0.58 ± 0.04	3.67 ± 0.43	6.25 ± 0.30	3.99 ± 0.32
HCHF+MCAO+PMG 50	0.52 ± 0.02^∗^	4.09 ± 0.33^∗^	14.90 ± 1.36^∗∗^	5.77 ± 0.96^∗^
HCHF+MCAO+PMG 100	0.51 ± 0.04^∗^	5.17 ± 0.86^∗∗∗^	15.54 ± 3.02^∗∗^	6.54 ± 1.68^∗∗∗^
HCHF+MCAO+PMG 200	0.51 ± 0.03^∗^	4.37 ± 0.37^∗∗^	6.76 ± 2.13	5.75 ± 0.48^∗^

Data are presented as mean ± SEM (*n* = 6/group). ^aaa^*p* values < 0.001; compared between naïve control rats which received normal diet and vehicle, and metabolic syndrome+MCAO rats which received HCHF, MCAO, and vehicle, ^bbb^*p* values < 0.001; compared between sham rats which received HCHF, sham operation, and vehicle, and metabolic syndrome+MCAO rats which received HCHF, MCAO, and vehicle and ^∗^, ^∗∗^, ^∗∗∗^*p* values < 0.05, 0.01, and 0.001, respectively; compared to metabolic syndrome+MCAO rats which received HCHF, MCAO, and vehicle. ND: normal diet; HCHF: high-carbohydrate high-fat diet; MCAO: middle cerebral artery occlusion; vitamin C: the Vitamin C at a dose of 250 mg.kg^−1^ BW; piracetam: the piracetam at a dose of 250 mg.kg^−1^ BW; PMG 50, PMG 100, and PMG 200: the phytosome containing the combined extract of mulberry fruit and ginger at doses of 50, 100, and 200 mg.kg^−1^ BW, respectively.

## Data Availability

I confirm that data are available and will be provided on request because during this period, all data are in the process of petty patent registration.
